# Bioequivalence Study of Two Oral Methocarbamol Formulations in Healthy Subjects Under Fasting Conditions: A Randomized, Open-Label, Crossover Clinical Trial

**DOI:** 10.3390/ph18030354

**Published:** 2025-03-01

**Authors:** Ana Ascaso-del-Rio, Paola Camargo-Mamani, Inmaculada Gilaberte, Mónica Díez-Hochleitner, Leonor Laredo-Velasco, Teresa Iglesias-Hernangómez, María Rosario Salas-Butrón, Laura Galán Caballero, Iván Alejandro Díaz-Rengifo, Carla Pérez-Ingidua, Emilio Vargas-Castrillón, Antonio Portolés-Pérez

**Affiliations:** 1 Clinical Pharmacology Department, Hospital Clínico San Carlos, C/Prof Martín Lagos s/n, 28040 Madrid, Spain; ana.ascaso@salud.madrid.org (A.A.-d.-R.); leonor.laredo@salud.madrid.org (L.L.-V.); tiglesias@salud.madrid.org (T.I.-H.); mariadelrosario.salas@salud.madrid.org (M.R.S.-B.); lgalancaballero@salud.madrid.org (L.G.C.); carla.perez@salud.madrid.org (C.P.-I.); emilio.vargas@salud.madrid.org (E.V.-C.); antonio.portoles@salud.madrid.org (A.P.-P.); 2 Instituto de Investigación Sanitaria del Hospital Clínico San Carlos, C/Prof Martín Lagos s/n, 28040 Madrid, Spain; ivanalejandro.diaz@salud.madrid.org; 3 Pharmacology and Toxicology Department, School of Medicine, Universidad Complutense de Madrid (UCM), 28040 Madrid, Spain; 4 Clinical Research Department, Faes Farma, Av. Autonomía 10, 48940 Leioa, Spain; igilaberte@faes.es (I.G.); mdiezh@faes.es (M.D.-H.)

**Keywords:** Robaxin, methocarbamol, bioequivalence, bioequivalence study, methocarbamol pharmacokinetics, methocarbamol bioequivalence

## Abstract

**Objective:** This study aimed to assess the bioequivalence of two oral methocarbamol formulations, as follows: the test (T) methocarbamol 1500 mg tablets and the reference (R) Robaxin^®^ 500 mg tablets (3 tablets, total dose: 1500 mg) under fasting conditions, and compare their pharmacokinetic performance. **Methods:** This was a single-center, phase I, randomized, open-label (blinded for analytical determination), two-sequence, two-period, crossover, bioequivalence study. A total of 32 healthy volunteers were randomly assigned to receive the T-R or R-T administration sequence. Each volunteer received a single dose of each methocarbamol formulation (T or R) separated by a washout period of 7 days. To evaluate the pharmacokinetic profile, blood samples were collected at nineteen time points after dosing. **Results:** The arithmetic mean C_max_ was 31.72 µg/mL for R and 32.39 µg/mL for T, and the arithmetic mean AUC_0−t_ was 90.25 h × µg/mL and 89.72 h × µg/mL, respectively. All adverse events reported were mild for both formulations. The 90% confidence intervals for the corresponding logarithmically transformed geometric mean ratios of C_max_ and AUC_0−t_ fell within the acceptance interval of 80.00–125.00%, as their values were 91.67–112.47% for ln(C_max_) and 92.34–103.47% for ln(AUC_0−t_). **Conclusion:** Therefore, one tablet of methocarbamol 1500 mg was found to be bioequivalent to the Robaxin^®^ 500 mg tablets (3 tablets), with comparable tolerability and safety profiles.

## 1. Introduction

Methocarbamol is a skeletal muscle relaxant that acts centrally in the nervous system [1,2]. It was approved for the treatment of muscle spasms in 1957 [2]. Methocarbamol is used as a short-term adjunct to symptomatic treatment of acute musculoskeletal disorders associated with painful muscle spasms. However, it has been used off-label for other painful conditions such as acute and chronic non-specific low-back pain, inflammatory arthritis, and fibromyalgia [1,3,4]. Methocarbamol is part of the pharmacotherapeutic group of centrally acting muscle relaxants and carbamic acid esters (ATC code: M03BA03) [5,6].

The mechanism of action of methocarbamol in humans has not yet been established, but it may be due to central nervous system depression [1]. It inhibits polysynaptic reflex transmission, predominantly at the spinal level [7,8]. It does not directly affect the contractile mechanism of the striated muscle, motor end plate, or nerve fiber, and there is no depression in motor activity [1,2]. Methocarbamol can act as an anticholinergic inhibitor of the midbrain reticular activating system, inducing depressed polysynaptic reflexes and decreasing muscle tone [1,9].

The recommended oral daily dose in adults (Robaxin^®^ 500 mg) is between 4 g and 6 g divided into three or four doses (8 to 12 tablets per day, divided into three or four doses). In severe cases, a higher dose may be necessary, with the maximum recommended dose being 7.5 g per day for the first 48–72 h (15 tablets per day) [6].

After oral administration, methocarbamol is rapidly absorbed [10]. It can be detected in the blood 10 min after intake [10]. Metabolism primarily occurs through dealkylation, hydroxylation, and conjugation in the liver, and within 72 h, 97–99% of the dose is excreted in the urine, mainly as water-soluble glucuronide and sulfate conjugates [10], with only a small proportion appearing in the stool. A trace amount of unchanged methocarbamol is also eliminated in the urine [10].

It is described that medication nonadherence can be intentional when polypharmacy and high pill burden become overwhelming for patients [11]. Actually, studies evaluating treatment adherence revealed that the main causes of poor compliance are associated significantly with younger age and with taking more tablets per day [12,13]. Therefore, a single oral dose of 1500 mg, although it is of larger size, is expected to improve patient compliance, particularly for polymedicated patients or those with complex treatment schedules, and is more likely to help to avoid medication errors.

The present study aimed to assess the bioequivalence of the reference drug (R) Robaxin^®^ 500 mg (3 tablets) compared with a new formulation of the test (T) drug methocarbamol 1500 mg (1 tablet) [14,15,16].

## 2. Results

### 2.1. Demographic Characteristics

**Figure 1:**
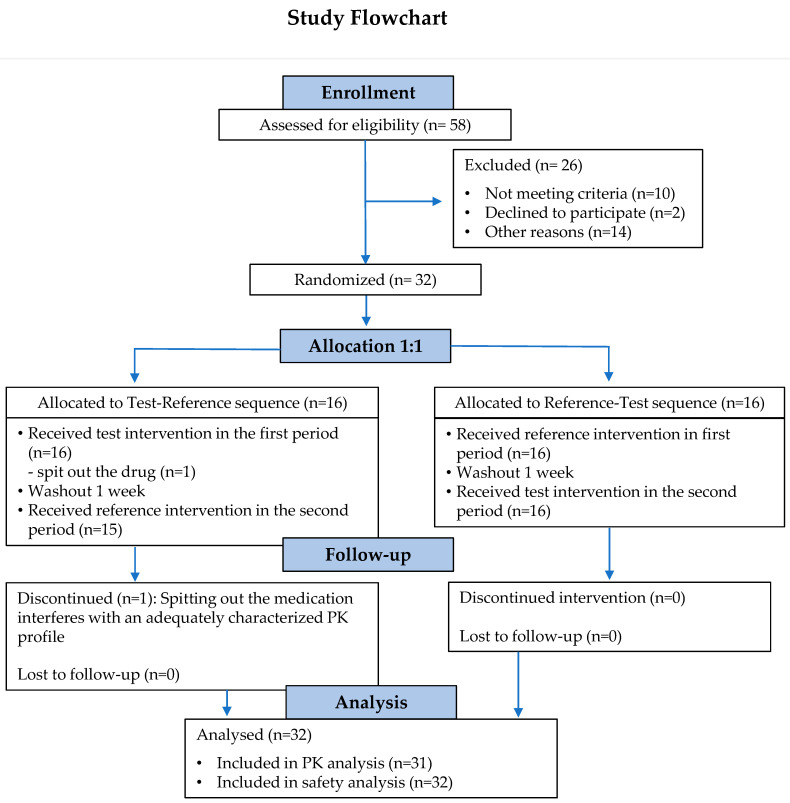
Study flowchart. *n*: number of subjects; PK: pharmacokinetic.

Fifty-eight (58) subjects were enrolled in the screening phase of the study. Among them, 32 subjects (15 males [46.87%] and 17 females [53.13%]) met all the inclusion criteria and none of the exclusion criteria for participation. None of the 32 subjects (100%) in the randomized population analysis set were excluded from the safety population. However, one subject (3.1%) in the T-R sequence group spat out part of the drug (T) and was excluded from the pharmacokinetic analysis (Figure 1).

For the randomized population (*n* = 32), all subjects were Caucasians, the rest of demographic and baseline characteristics are shown in Table 1.

### 2.2. Pharmacokinetics

**Figure 2:**
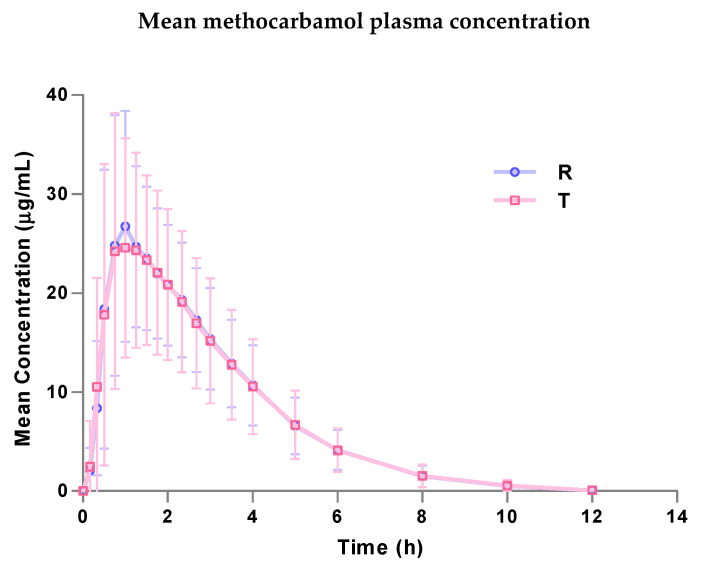
Mean methocarbamol plasma concentration (µg/mL)/time (h) curve (±SD) for reference (R) and test (T) formulations. The graph shows the arithmetic mean concentrations at each sampling point, along with the variability (maximum and minimum concentrations). Note that the maximum concentrations in the graph do not represent the mean C_max_ values.

The methocarbamol plasma concentrations are represented with respect to sampling times after each treatment (Figure 2). The plasma concentrations were below the limit of quantification (LOQ) before dose administration in all subjects during both periods. The concentrations remained above the LOQ in all subjects for 8 h from administration, returning to being below the LOQ overall after the first 10 h. The concentration–time profiles of the two formulations were similar. The overall mean maximum concentration (C_max_) was 31.72 µg/mL for the R formulation and 32.39 µg/mL for the T, and the geometric means were 29.96 µg/mL and 30.51 µg/mL, respectively. It is worth noting that the maximum concentrations shown in Figure 2 at specific time points do not correspond to the mean C_max_ values (32.39 ng/mL for the test and 31.72 ng/mL for the reference). This discrepancy arises because the mean C_max_ values are calculated as the average of individual C_max_ values across all subjects, each of which may occur at different time points depending on the individual pharmacokinetic profiles. Figure 2, on the other hand, represents the average methocarbamol plasma concentration at each scheduled sampling point, along with the observed variability (maximum and minimum values). The mean area under the curve from 0 to the last measurable concentration (AUC_0−t_) was 90.25 h × µg/mL for the R and 89.72 h × µg/mL for the T, and the geometric means were 84.75 h × µg/mL and 83.07 h × µg/mL, respectively. Both parameters showed a very high degree of similarity between the formulations.

Furthermore, the logarithmically transformed AUC_0−t_ and C_max_ ratios between formulations (ln AUC_0−t_ [T/R]; ln C_max_ [T/R]) were within the reference bioequivalence interval of 80–125% established for a CI of 90% (Table 2).

The within-subject variability, (CV%) was 24% for C_max_ and 13% for AUC_0–t_.

Regarding other pharmacokinetic parameters, the R formulation showed a mean time to reach C_max_ (T_max_) of 1.07 h, while the T formulation resulted in a mean T_max_ of 1.15 h. The median T_max_ was 1.00 h for both formulations. The elimination half-lives showed similar values for both formulations (geometric means of 1.38 h for R and 1.40 h for T), along with the remaining secondary variables, which were analyzed descriptively (Table 3).

### 2.3. Clinical Observations and Analysis of Safety and Tolerability

All 32 randomized subjects received at least one oral dose of methocarbamol; therefore, all of them were included in the safety population. In total, 63 exposures took place, including 32 with the T and 31 with the R formulation. Overall, 27 adverse events (AEs) were recorded in 14 subjects. A total of 10 events were reported after the most recent treatment with T, and 17 events were reported after the most recent treatment with R. A total of 18 AEs reported in 12 subjects were related to the treatment, with 11 occurring during the treatment period with the R and 7 following the administration of the T. All AEs were mild, and none led to premature study termination. Serious adverse events were not observed. The summary of adverse events is listed in Table 4.

The most frequently reported AE was headache, with nine cases in nine subjects, all of which were mild and possibly related to the administration of methocarbamol. For six of these cases, the last formulation received was Robaxin^®^ (R).

No clinically significant anomalies were detected in the follow-up visits compared to the baseline values in physical exploration, vital signs, or electrocardiogram (ECG). Only one clinically significant alteration (one event of hyperemic pharynx) in one subject was detected and evaluated as being unrelated to the treatment. Compared to the baseline ECG findings, some isolated cases of asymptomatic sinus bradycardia and early repolarization signs were found, both common among healthy young people and not clinically significant. In blood analysis, no clinically significant analytical alterations were detected. In urianalysis, two cases of asymptomatic bacteriuria were detected and resolved without sequelae.

## 3. Discussion

This single-center, phase I, randomized, open-label, crossover, bioequivalence study was designed to evaluate the bioequivalence of the following two methocarbamol formulations: methocarbamol 1500 mg (one tablet) and Robaxin^®^ 500 mg (3 tablets, total dose 1500 mg), in healthy volunteers under fasting conditions. The study adhered to the protocol and data management and was performed with rigorous quality control checks. Only minor protocol deviations occurred.

The strength was chosen according to the current guidelines [14] and considering the safety profile [6,17]. Consequently, the highest single dose of methocarbamol (1500 mg) was selected [6] to be tested against the same dose of the reference product (3 tablets of 500 mg).

The new methocarbamol 1500 mg formulation would represent an improvement by reducing the dosage regimen from three 500 mg tablets to a single tablet, offering significant advantages, including improved patient compliance and adherence. Medication adherence is defined, according to the World Health Organization, as “the extent to which a person’s behavior—aking medications, following a diet, and/or executing lifestyle changes corresponds with agreed recommendations from a healthcare provider” [18]. It is described that medication nonadherence can be intentional when polypharmacy and high pill burden become overwhelming for patients [11,19]. Studies evaluating treatment adherence revealed that the main causes of poor compliance are associated significantly with younger age and with taking more tablets per day [12,13,19]. Since medication adherence is critical for effectively managing the patient’s disease, developing different strategies to improve adherence is important. Among the various approaches investigated to improve adherence, significant positive results have been found, among others, in studies utilizing strategies to simplify medication regimens. Simplification can include discontinuing unnecessary (or less necessary) medications, decreasing the frequency of medication administration, reducing the overall pill burden, and using single-pill combination therapies [20].

A narrative review performed in 2020 aimed to describe and critically evaluate medication adherence interventions published between 2017 and 2020 for patients with hypertension [21]. Across the 42 studies analyzed, 19% tested the effectiveness of simplifying the medication regimen, and all reported statistically significant improvements in medication adherence attributable to the intervention [21]. Five studies reported improved medication adherence by reducing the number of daily pills [22,23,24,25,26]. The ALL-IN-ONE trial aimed to compare the efficacy and safety of a once-daily fixed combination versus a free-drug combination of three antihypertensive agents and a statin, showing that the once-daily fixed combination was associated with greater compliance and better cardiovascular risk control [27]. Also, in the large SECURE trial, in which 2499 European patients were randomly assigned to a polypill, high levels of adherence were more commonly reported in the polypill group compared with usual care (74.1% versus 63.2%) [28]. A systematic literature review investigated the impact of single-pill-combinations (SPC) compared to free equivalent combination (FEC) on adherence and persistence in patients with hypertension, dyslipidemia, or both. SPCs were associated with significantly improved adherence, assessed through a medication-possession ratio of ≥80% (odds ratio (OR) 0.42, *p* < 0.01) and proportion of days covered of ≥80% (OR 0.45, *p* < 0.01) [29].

A 24-month prospective study aimed to determine adherence, efficacy, and safety after conversion of stable liver transplant (LT) recipients from a standard twice-daily immediate-release Tacrolimus (IR-Tac) to a novel once-daily life cycle pharma Tacrolimus (LCP-Tac) formulation [30]. The overall adherence to the BAASIS© questionnaire for self-reported adherence increased by 57% until month 24, compared to the baseline (51% vs. 80%) [30]. A large-scale meta-analysis and subgroup analysis was performed in 2023 to compare the effect of fixed-dose combination (FDC) therapy with that of FEC therapy on medication adherence [31]. A total of 61 studies were finally included. The most frequent disease type was hypertension (34 studies), followed by diabetes (9 studies), acquired immune deficiency syndrome (AIDS) (5 studies), hyperlipidemia (2 studies), and dyslipidemia (2 studies). The results showed that, compared to FEC, FDC significantly improved the medication compliance of patients by 1.29 times (95% CI: 1.23–1.35, *p* < 0.00001) [31]. In patients with type 2 diabetes mellitus (T2DM) and cardiovascular disease, the adherence to treatment was 19% and 28% higher, respectively, in the FDC group than in the FEC group (*p* < 0.00001) [31].

All of the above demonstrates that, in different types of diseases, reducing the number of pills and, thus, simplifying medication regimens improves patient adherence and compliance and improves the control of the disease. Thus, the new methocarbamol 1500 mg formulation would represent an improvement by reducing the dosage regimen to a single tablet, simplifying treatment management by reducing the pill burden, which would improve patient compliance.

The new methocarbamol 1500 mg formulation is larger (22 mm × 11.5 mm) than the reference 500 mg formulation (12 mm × 6.6 mm). The size and shape of the preparations can be challenging for some patients, leading to difficulties in swallowing and, consequently, suboptimal medication adherence, especially for older people [32,33]. A long diameter of 21.5–22 mm has been identified as causing swallowing difficulties in patients [33,34], and the new 1500 mg formulation presents a similar size. In addition, between 10 and 40% of adult patients find tablets and/or capsules difficult to swallow [33,35,36]. With all of this in mind, the new 1500 mg formulation presents a score line to facilitate breaking for ease of swallowing. Thus, in this way, we maintain the simplification of the treatment regimen and provide a solution in the hypothetical case that the patient has difficulty swallowing.

The 7-day wash-out period was far longer than five half-lives, and the pre-dose samples did not exhibit any detectable drug level, ensuring no carryover effect between each study dose administration.

The pattern of plasma concentrations over time was similar for both formulations, indicating stability and reliable measurements throughout the study. The concentration–time profiles were also very similar for both the test methocarbamol 1500 mg and the reference Robaxin^®^ 3 × 500 mg.

The bioavailability parameters of the analytes were described and compared between formulations and were found to be similar. According to the current regulations of the EMA for bioequivalence [14], two formulations in a non-compartmental model are bioequivalent when the 90% confidence interval for the ratio of the test and reference products for the pharmacokinetic parameters AUC_0−t_ and C_max_ fall within the acceptance interval of 80.00–125.00%. Consequently, the test methocarbamol 1500 mg and the reference Robaxin^®^ 500 mg (three tablets) are considered bioequivalent. Both the R and T formulations showed the same median T_max_ values. The means and geometric means of C_max_, AUC_0−t_, and plasma concentration half-life (T_½_β) were similar for both formulations, indicating comparable elimination rates. The same happened with the area under the plasma concentration curve extrapolated to infinite time (AUC_0–∞_) means and geometric means, which were consistent with the T_½_β and C_max_ findings, thereby supporting the comparability of the formulations.

The values observed for the residual area also indicated that both formulations exhibited a low residual area, suggesting that the sampling time points adequately captured the drug concentration profile.

Both formulations of methocarbamol were safe and well tolerated, all AEs detected were mild, and the safety profiles of both formulations were comparable.

These results are consistent with those reported by Schlegelmilch et al. (2008) [2], who compared the same total dose (1500 mg) of two methocarbamol formulations (2 × 750 mg tablets versus 2 × 750 mg tablets) in the same number of healthy subjects (*n* = 32) under fasting conditions. In both studies, the geometric mean ratios (GMRs) for C_max_ and AUC_0−t_ fell within the regulatory acceptance range of 80.00–125.00% for bioequivalence. Additionally, the within-intrasubject variability (CV%) observed for C_max_ 24% was comparable to that of the previous study (24.8%), both of which are below the 30% threshold [2]. According to current EMA bioequivalence guidelines, a CV% below 30% is not considered indicative of a highly variable drug [14]. This finding suggests consistency between the two studies, supporting the idea that the formulations exhibit similar pharmacokinetic profiles. It is also noteworthy that the low variability observed enhances the bioequivalence assessment’s reliability, reinforcing the formulations’ interchangeability.

As a limitation, the study was performed only on healthy Caucasian subjects under fasting conditions, which could not represent all the possible scenarios (e.g., different races, patients, or under feeding conditions). Nevertheless, methocarbamol has been used for the treatment of muscle spasms since 1957 [2]; therefore, its profile is well-known, and the new formulation can be reasonably expected to hold true in other populations. The strengths include the blinding for the analytical determination, the crossover design, and the fact that all participants showed similar health status.

While the observed variability in pharmacokinetic parameters is consistent with previously reported data and does not indicate high within-subject variability (CV < 30%), a limitation of this study could be the lack of a detailed analysis of potential correlations between pharmacokinetic parameters and demographic or anthropometric characteristics, such as BMI categories (<25 and ≥25–30), age, or sex, that could influence the dispersion of pharmacokinetic parameters, however, their analysis was considered beyond the scope of this research.

Currently, no additional studies evaluating the bioequivalence of the individual 500 mg or 1500 mg tablet formulations are planned, as this study was specifically designed to compare these two formulations and support their interchangeability.

## 4. Materials and Methods

This study was performed in the Clinical Pharmacology Studies Unit in the Hospital Clínico San Carlos in Madrid, Spain. This study was conducted in accordance with the Declaration of Helsinki, Good Clinical Practice guidelines, and applicable local laws and regulations. The study results and protocol are not publicly available in the EudraCT registry, as phase I trials conducted solely on adults and that are not part of an agreed Pediatric Investigation Plan (PIP) are not subject to public disclosure. This study was designed following EMA bioequivalence guidelines to ensure that the new formulation meets the same therapeutic standards as the reference product. EMA guidelines stipulate that bioequivalence studies should employ a randomized crossover design in healthy volunteers, demonstrating that the 90% confidence intervals for the geometric mean ratios of C_max_ and AUC_0−t_ fall within the acceptance range of 80.00–125.00%. Additionally, the study must utilize validated analytical methods and comply with Good Clinical Practice standards [14]. This study (code 22/149-EC_M) obtained approval from the local independent ethics committee at the Hospital Clínico San Carlos, and all patients provided written informed consent before participating. The study was also approved by the Spanish Agency of Medicines and Health Products (Agencia Española de Medicamentos y Productos Sanitarios—AEMPS) and was registered on the European Clinical Trials Register (EudraCT) under the identifier EudraCT Number 2021-006466-21.

All subjects freely agreed to participate in this study and provided written consent to participate in the clinical trial after receiving and understanding information about the study design, objectives, possible derivative risks, and the right to deny their collaboration at any moment.

### 4.1. Study Population

Healthy volunteers were recruited and examined after signing the informed consent forms, and those meeting all of the inclusion criteria and none of the exclusion criteria were finally randomized. These subjects were healthy male or female individuals aged between 18 and 50 years (both included), with a body weight between 50 and 100 kg and a BMI of 18.5–30 (BMI = weight (kg)/height (m^2^)) and no relevant medical history. Their physical examination, vital signs, blood and urine analysis, and electrocardiography results during screening were normal or abnormal, not clinically significant. The subjects had no plans to give birth or become pregnant and voluntarily used an effective contraceptive method.

The main exclusion criteria were as follows: pregnancy, lactation, or planning a pregnancy for female participants; smoking; history of drug abuse or alcoholism or positive result for abuse substances in screening tests; use of any medication or substance that could interfere with the study aim; participation in a clinical trial within 3 months prior to inclusion; history of significant medical condition; any clinically significant deviation from normal in the physical or ECG examinations or medically significant values outside of the normal range in clinical laboratory tests; positive results for HIV or active SARS-CoV-2 (if performed); hepatitis B or C; and current diseases that can alter drug absorption, distribution, metabolism, and/or excretion. The complete list of the inclusion and exclusion criteria is provided in Supplementary Tables S1 and S2.

Furthermore, subjects should not consume food or beverages containing grapefruit within seven days before each treatment period or during the study. They could not consume large amounts of drinks/xanthines (equivalent to 400 mg of caffeine per day) or alcohol within two days before each treatment period and during each sample collection period. The volunteers were informed that they were not allowed to perform strenuous physical exercise within 72 h before the first administration and during each pharmacokinetics sample period. On each admission day, investigators confirmed that the subjects adhered to the abovementioned restrictions, that all inclusion criteria were met, and that none of the exclusion criteria were fulfilled. This information was recorded in the CRF.

### 4.2. Sample Size

The sample size was calculated based on the estimated intra-subject variability of C_max_ obtained from a previous study [2], which appeared to be around 24%. Consequently, the minimum number of subjects required to achieve the bioequivalence range of 80.00–125.00% with a statistical power of at least 90%, an alpha error of 0.05, accounting for potential dropouts, and a formulation ratio of 0.95 was estimated to be 32 subjects. Calculation tables for bioequivalence crossover studies [37] were used to determine the estimation.

### 4.3. Study Formulation

The test product (methocarbamol 1500 mg tablets) is an oral formulation consisting of one tablet containing 1500 mg of methocarbamol. One tablet was administered as a single dose. The reference product (Robaxin^®^ 500 mg tablets) is an oral formulation consisting of one tablet containing 500 mg of methocarbamol. Three tablets (total dose 1500 mg) were administered as a single dose. Both formulations are manufactured, marketed, and provided by FAES FARMA., S.A., Madrid, Spain.

### 4.4. Study Design—Randomization and Administration Process

This was a single-center, phase I, randomized, open-label (blinded for analytical determination), two-sequence, two-period, crossover, bioequivalence study in which a single oral dose of each formulation (test [T]—one tablet of methocarbamol 1500 mg—and reference [R]—Robaxin^®^ 500 mg, three tablets) was administered under fasting conditions.

**Figure 3:**
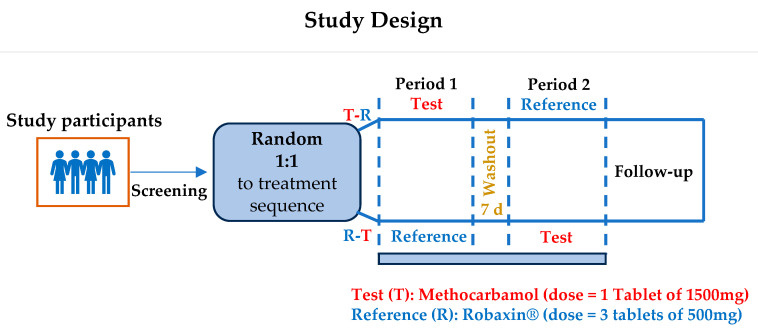
Study design. d: days.

The study design (Figure 3) included a screening period (3 weeks) and an experimental phase of <3 weeks that included two treatment periods (each of them of around 22 h) with at least one week-long washout period between them. In each period, a single dose of each formulation was administered. Follow-up visits were conducted 7 days after the last treatment. The subjects were randomized in a ratio of 1:1 to one of the two treatment sequences (16 to T-R sequence and 16 to R-T sequence). The subject’s allocation to treatments was assigned according to the randomization list determined by computer-generated blocked randomization to ensure that the number of subjects distributed to each treatment sequence would not differ (blocks of 4 subjects).

The participants attended the Clinical Trial Unit the day before the first and second drug administration and remained hospitalized for approximately 22 h. Upon admission, the volunteers received a standard supper and then remained under fasting conditions until at least 4 h after receiving the treatment the following day. The next morning, at 8:00 h, the treatment was orally taken with 200 mL of water after having fasted for at least 8 h. The medication was administered under medical supervision, and either the investigator or an authorized delegate was responsible for checking the mouths of the subjects after intake of the study medication to ensure that the participants had swallowed the drug. The subjects were allowed to drink water “ad libitum,” except 1 h before and 2 h after treatment administration. The participants received standardized meals at 14:30 h and 17:30 h.

### 4.5. Sample Collection and Analytical Method

Several blood samples (6 mL) were obtained per subject and period via a venous catheter at each of the following time points (in hours) during each study period: 0 (basal sample), 0.10, 0.20, 0.30, 0.45, 1, 1.15, 1.30, 1.45, 2, 2.20, 2.40,3, 3.30, 4, 5, 6, 8, 10, and 12. Baseline samples were collected before drug administration. The number of samples and time points were calculated considering methocarbamol’s pharmacokinetic characteristics and the regulatory guidelines on bioavailability and bioequivalence [1,12,14,16].

The blood samples were collected at room temperature in EDTA K2 tubes and immediately put into an iced pack container then centrifuged at 4–8 °C at 1900 g for 10 min. The whole process was completed in less than 1 h after blood collection. The plasma samples were divided into test and backup aliquots, with each tube containing at least 1 mL (minimum volume required/aliquot was 0.5 mL of plasma), and frozen at −80 °C (±15 °C). To maintain blinding of the analyst responsible for measuring methocarbamol plasma concentrations, the test samples were sent to an external testing laboratory labeled with a QR code. Shipping was conducted at −80 °C (±15 °C) on dry ice, with temperature monitoring. Backup samples were stored at the Clinical Trials Unit in a −80 °C refrigerator if reanalysis was necessary.

Plasma concentrations of methocarbamol were analyzed using a fully validated method following the current guidelines of high-performance liquid chromatography-tandem mass spectrometry (LC/MS/MS), in accordance with the principles of Good Laboratory Practice (GLP). The calibration model used was linear with a 1/X^2^ weighting factor, without forcing the intercept to zero, the concentration range was 0.5–50 μg/mL, and the LOQ was established at 0.5 µg/mL, considering concentrations lower than LOQ as zero. The correlation coefficient was set at r ≥ 0.9900. Quality control (QC) samples comprised 1.50 (QC1), 25.02 (QC2), 37.53 (QC3), 2.50 (QC4), and 75.05 (Low Diluted QC), with a dilution factor of 2. Details of the analytical method and calibration have been included in the Supplementary Materials.

Methocarbamol chromatographic separation was performed using a Zorbax SB-C18, 4.6 × 50 mm, 3.5 μm (Agilent Technologies) column under isocratic conditions with a 1.00 mL/min flow rate. Methocarbamol was detected using an API 4000 (Sciex) mass spectrometer (Sciex). The mass transitions for methocarbamol and methocarbamol-d5 were m/z 242.09 → 118.00 and m/z 247.09 → 123.00, respectively.

Data acquisition and integration were performed using validated analyst version 1.6.2 software (Sciex). The study sample concentrations were calculated automatically using chromatographic software.

### 4.6. Pharmacokinetic Analysis

A non-compartmental analysis model was performed using Phoenix^®^ WinNonlin^®^ software version 8.3 (Pharsight Certara Corporation, Princeton, NJ, USA) for the various pharmacokinetic parameters. The primary analysis compared differences in bioavailability between the two formulations. All pharmacokinetic parameters were obtained from plasma concentrations of methocarbamol.

The primary variables to be analyzed for bioequivalence were the C_max_ and the AUC_0−t_ calculated using the linear trapezoidal rule method until the last measurable concentration. The mean concentration vs. time at each scheduled sampling point was represented graphically (calculated as the mean of the methocarbamol plasma concentrations obtained from all subjects at each sampling point). It can be observed that the maximum concentration at a specific time point does not correspond to the mean C_max_, as this variable is calculated independently of time.

The secondary pharmacokinetic parameters were as follows: T_max_; AUC_0–∞_, calculated as AUC_0−t_ + (Ct/Ke), where Ct is the last quantifiable concentration and Ke the terminal elimination constant; T_½_β, calculated as 0.693/Ke, where Ke was the elimination constant calculated from the slope of the semilogarithmic representation of the concentration against time for the final part of the curve; residual area, which is the fraction of AUC_0–∞_ corresponding to extrapolation from T_last_ to infinity (AUC_0–∞_ − AUC_0−t_/AUC_0–∞_*100); mean residence time of the product in the organism (MRT); the rate at which the active drug is removed from the body (Cl), calculated as drug elimination divided by the plasma concentration of the drug; and the apparent volume of distribution (Vd), i.e., the hypothetical fluid volume through which the drug is dispersed, calculated by dividing the total amount of drug given by the concentration of the drug in plasma. Continuous variables were analyzed as descriptive statistics and categorical variables as frequencies and percentages when applicable.

An analysis of variance (ANOVA) model incorporating the following factors was used to evaluate the multiplicative variables: subject, formulation, period, and treatment sequence. The results were presented using both the original and ln-transformed variables. The effects were assessed at a significance level of 5%. Bioequivalence was determined by comparing the T and R formulations using a parametric approximation for AUC_0−t_ and C_max_, with a 90% CI defined as the ratio after ln-transformation. The acceptance interval for bioequivalence was 80.00–125.00%.

### 4.7. Safety Assessment

Safety variables were secondary. Adverse events, concomitant medication, physical examination, vital signs (blood pressure, heart rate, and temperature), changes in laboratory results (hematology, urine, and blood biochemistry), urine pregnancy tests, abuse drug tests, and ECG were monitored throughout this study to ensure the safety of the subjects in the clinical trial.

Tabular listings and summary statistics were provided and reported descriptively using the validated statistical software SPSS^®^ release 26 (IBM Corp., Armonk, NY, USA) and Stata^®^ release 16.1 (StataCorp LLC, College Station, TX, USA). The clinical and safety data were prepared using an Access^®^ database.

## 5. Conclusions

The test formulation methocarbamol 1500 mg and the reference product Robaxin^®^ 500 mg (3 tablets) were bioequivalent in healthy subjects under fasting conditions. This conclusion was drawn because the 90% CI for the corresponding geometric mean ratios of C_max_ and AUC_0−t_ fell within the acceptance interval of 80.00–125.00%. Methocarbamol was well tolerated, and the safety profile of both formulations was comparable.

## Figures and Tables

**Table 1:** Categorical and quantitative demographic and baseline characteristics of the randomized population (*n* = 32).

Parameter	Categories			*n*		%
Sex	Female			17		53.1
	Male			15		46.9
**Parameter**		**Mean**	**SD**	**Min**	**Median**	**Max**
Age (years)	Overall	29.72	7.82	18.00	28.50	44.00
	Female	29.18	6.99	20.00	29.00	41.00
	Male	30.33	8.87	18.00	28.00	44.00
Weight (kg)	Overall	68.43	11.64	53.00	67.30	96.60
	Female	61.48	6.83	53.00	60.70	75.20
	Male	76.71	10.45	57.80	78.60	96.60
Height (cm)	Overall	167.13	10.62	143.00	165.50	183.00
	Female	159.35	7.75	143.00	159.00	177.00
	Male	175.93	4.96	165.00	178.00	183.00
BMI (kg/m^2^)	Overall	24.39	2.49	19.75	24.00	29.16
	Female	24.08	2.10	20.10	24.00	28.16
	Male	24.75	2.91	19.75	25.59	29.16

%: percentage; BMI: Body Mass Index; *n*: number of subjects; Max: maximum; Min: minimum; SD: standard deviation.

**Table 2:** Bioequivalence statistics, average C_max_, and AUC_0−t_ in pharmacokinetic population.

Dependent	Units	Ratio Test/Reference (%)	CI 90 Lower	CI 90 Upper
ln(C_max_)	µg/mL	101.54	91.67	112.47
ln(AUC_0−t_)	h × µg/mL	97.75	92.34	103.47

CI: confidence interval; ln: natural logarithm.

**Table 3:** Descriptive statistics of pharmacokinetic variables of methocarbamol.

Variable	Statistics	Test	Reference	Variable	Statistics	Test	Reference
**AUC_0−__t_** [h × µg/mL]	*n*	31	31	**Cl** [mL/h]	*n*	31	31
	Mean	89.72	90.25		Mean	18,971.37	18,421.72
	SD	36.70	31.88		SD	7242.45	6998.10
	GeoM	83.07	84.75		GeoM	17,664.19	17,280.03
	G_CV	41.37	38.07		G_CV	40.46	37.06
**C_max_** [µg/mL]	*n*	31	31	**T_½_β** [h]	*n*	31	31
	Mean	32.39	31.72		Mean	1.41	1.40
	SD	11.07	11.15		SD	0.23	0.25
	GeoM	30.51	29.96		GeoM	1.40	1.38
	G_CV	37.14	35.22		G_CV	16.69	18.43
**AUC_0–∞_** [h × µg/mL]	*n*	31	31	**MRT** [h]	*n*	31	31
	Mean	91.45	92.17		Mean	2.75	2.74
	SD	36.79	31.84		SD	0.47	0.38
	GeoM	84.92	86.81		GeoM	2.71	2.71
	G_CV	40.46	37.06		G_CV	18.27	14.52
**Residual area** [%]	*n*	31	31	**Vd** [mL]	*n*	31	31
	Mean	2.17	2.37		Mean	37,510.99	35,757.60
	SD	1.01	1.09		SD	12,366.56	10,562.80
	GeoM	1.98	2.13		GeoM	35,559.30	34,281.31
	G_CV	45.86	50.32		G_CV	34.48	30.31
**T_max_** [h]	*n*	31	31				
	Mean	1.15	1.07				
	SD	0.67	0.59				
	Min	0.50	0.50				
	Med	1.00	1.00				
	Max	3.50	3.00				

AUC_0−t_: area under the plasma concentration curve, calculated using the linear trapezoidal method until the last measurable concentration; AUC_0–∞_: area under the plasma concentration curve extrapolated to infinite time; Cl: clearance; C_max_: maximum plasma concentration; MRT: mean residence time; T_max_: time until C_max_ is reached; T_½_β: plasma concentration half-life; Vd: volume of distribution. Note: C_0_ for the R and T was 0.

**Table 4:** Summary of adverse events.

	Test Period (Treated = 32)	Reference Period (Treated = 31)	Total (Treated = 32)
Number of AEs/*n*			
Overall	10/7	17/10	27/14
Study-drug-related	7/7	11/7	18/12
**Type of study-drug-related AEs**			
Number of AEs/*n*			
Nervous system disorder ^(1)^	4/4	7/7	11/11
Gastrointestinal disorder ^(2)^	1/1	1/1	2/2
General disorders and administration site conditions ^(3)^	1/1	1/1	2/2
Musculoskeletal and connective tissue disorders ^(4)^	1/1	-	1/1
Skin and subcutaneous tissue disorders ^(5)^	-	2/1	2/1

AEs, adverse events; *n*, number of subjects with at least one AE. ^(1)^ Headache, dizziness. ^(2)^ Nausea, abdominal pain. ^(3)^ Feeling abnormal. ^(4)^ Muscular weakness. ^(5)^ Pruritus and rash.

## Data Availability

The data presented in this study are available upon request from the corresponding author due to confidentiality reasons.

## Supplementary Materials

The following supporting information can be downloaded at: https://www.mdpi.com/article/10.3390/ph18030354/s1, Table S1. Inclusion criteria; Table S2. Exclusion criteria. Table S3. Summary of calibration curve parameters. Figure S1. Sample of calibration plot along with the regression equation and the correlation coefficient (r).
