# Supplementary material for: Bioequivalence Study of Two Oral Methocarbamol Formulations in Healthy Subjects Under Fasting Conditions: A Randomized, Open-Label, Crossover Clinical Trial
Source: Pharmaceuticals (Basel). 2025 Mar 1;18(3):354. doi: 10.3390/ph18030354 (PMC11946590; doi:10.3390/ph18030354)
Supplement: Supplementary file 1 [file pharmaceuticals-18-00354-s001.zip › pharmaceuticals-3445235-supplementary.pdf]

1. Supplementary material: eligibility criteria

Table S1. Inclusion criteria

INCLUSION CRITERIA
1. Healthy male or female subjects aged between 18 and 50 years (both included).
2. Subjects who give their written consent to participate in the clinical trial after receiving information including the study design, the project's objectives, the possible derivative risks, and the right to deny their collaboration at any moment.
3. Clinical history and physical examination within normality.
4. Vital signs and electrocardiography register within normality.
5. Body weight 50-100 kg and body mass index (BMI) 18.5- 30 [BMI = weight (kg)/ height^2 (m^2)].
6. For females of childbearing potential: willingness to perform pregnancy tests and use a medically acceptable barrier method of contraception throughout the study, from at least 4 weeks previous to the IMP intake until one week after the study ends (when applicable). The following hormonal contraceptives are also allowed: levonorgestrel, ethinyl estradiol, drospirenone, etonogestrel, norgestimate, norelgestromin, dienogest and gestodene. Intrauterine hormone-releasing systems (IUS), bilateral tubal occlusion, vasectomised partner (provided that partner is the sole sexual partner of the clinical trial participant) or sexual abstinence (if defined as refraining from heterosexual intercourse during the entire period of risk associated with the clinical trial treatment), are also acceptable contraceptive methods. The investigator is responsible for determining whether the subject has adequate birth control for study participation.
7. For males with female partners of childbearing potential: acceptance to use birth control method (condom with or without spermicide) throughout the study duration, from the time of informed consent signature until one week after the study ends (when applicable).

For the selection of a subject all the answers should be affirmative.

Table S2. Exclusion criteria

EXCLUSION CRITERIA
1. Pregnancy, lactation or planning a pregnancy.
2. Smoking within six weeks prior to study inclusion and during the study.
3. History of drug abuse or alcoholism and/or use of any commonly abused drugs within a month prior to study entry, or positive result to abuse substances in screening tests.
4. Alcohol consumption within 48 hours prior to admission and during each pharmacokinetics samples period.
5. High consumption of stimulating drinks/xanthines (equivalent to 400 mg of caffeine per day; a cup of coffee approximately containing 100 mg of caffeine) within 48 hours prior to admission and during each pharmacokinetics samples period.
6. Strenuous physical exercise within 72 hours prior to admission and during each pharmacokinetics samples period.
7. Not having received a full vaccination course against SARS-CoV-2, according with the criteria at the time of inclusion.
8. Use of any medication or substance that can interfere with the study aim:<br>* Regular use of potentially interfering medication within two weeks prior to admission for treatment (such as anaesthetics or appetite suppressants)<br>* Use of enzyme inhibitors or inducers within at least 5 half-lives prior to inclusion (barbiturates, carbamazepine, erythromycin, phenytoin, etc). In the case of grapefruit, consumption is prohibited within 7 days prior each treatment period and during the study.<br>* Any concomitant medication evaluated as interfering medication by the investigator or the sponsor, before including the volunteer.
9. Participation in a clinical trial within 3 months prior to inclusion or participation in 4 clinical trials during the last year.
10. History of significant medical condition in the investigator's opinion or major surgery in the past 3 months up to inclusion.
11. Any clinically significant deviation from normal in the physical or ECG examinations or medically significant values outside the normal range in clinical laboratory tests.
12. Subjects with known contraindications, such as comatose status or pre-coma, known cerebral pathology, history of seizures or epilepsy, or myasthenia gravis, or sensitivities to the use of the investigational medicinal products (IMPs) or any of their components.
13. Positive results for HIV or active SARS-CoV-2 (if performed), hepatitis B or C.
14. Current diseases which can alter drug absorption, distribution, metabolism and/or excretion (malabsorption, oedemas, liver or renal impairment, etc).
15. Blood loss or blood donation (over 200 ml within 3 months prior to study inclusion).
16. Blood and blood products transfusion in the past 6 months.
17. Inability or unwillingness to cooperate with the investigators.
18. Any reason in the investigator's opinion that could interfere in the subject's participation in the study.

For the selection of a subject all the answers should be negative.

2. Supplementary material: validation method

Validation of an Analytical Method for the Determination of Methocarbamol in Human EDTA K2 Plasma by LC/MS/MS

(Information extracted from the validation repot)

The aim of this study was the full validation of an analytical method for the determination of methocarbamol in human EDTA K2 plasma.

The method validation was performed according to the study plan and the current Guidelines detailed below:

- Guideline for Good Clinical Practice E6(R2), Step 5, European Medicines Agency (EMA), December 2016. (EMA/CHMP/ICH/135/1995). Adopted by CHMP on 15 December 2016.
- Reflection paper for laboratories that perform the analysis or evaluation of clinical trial samples, European Medicines Agency (EMA), February 2012. (EMA/INS/GCP/532137/2010).
- Guideline on Bioanalytical Method Validation, European Medicines Agency (EMA), July 2011. (EMEA/CHMP/EWP/192217/2009 Rev. 1 Corr. 2**).
- Guideline on the Investigation of Bioequivalence, European Medicines Agency (EMA), January 2010. (CPMP/EWP/QWP/1401/98 Rev. 1/Corr**).
- Good Clinical Laboratory Practice (GCLP), World Health Organization (WHO), 2009. (ISBN 978 92 4 159785 2).
- FDA Guidance for Industry: “Bioanalytical Method Validation”, U.S. Department of Health and Human Services, Food and Drug Administration, Center for Drug Evaluation and Research (CDER), Center for Veterinary Medicine (CVM), May 2018, Rockville, MD.
- All applicable regulations, including the recommendations laid down in the most recent version of the Declaration of Helsinki.
- Good Laboratory Practice (GLP) regulations: OECD Principles of Good Laboratory Practice revised in 1997 (ENV/MC/CHEM(98)17), 2004/10/EC Directive, February 11th 2004 and Royal Decree 822/1993 of May 28th modified by the 1369/2000 of July 19th.

Table S3 Summary of calibration curve parameters

Analyte: **Methocarbamol**

Biological Matrix: **Human EDTA K_2 plasma**

Batch Results Table No.	Slope	Y-Intercept	Correlation Coefficient (r)
001-3735V	0.192	0.00424	**0.9998**
002-3735VR1	0.196	0.00451	**0.9997**
003-3735V	0.195	0.00899	**0.9996**
006-3735V	0.192	0.00950	**0.9995**
N	4	4	4

Acceptance criteria:

$r \geq 0.9900$

Figure S1. Sample of calibration plot along with the regression equation and the correlation coefficient (r)

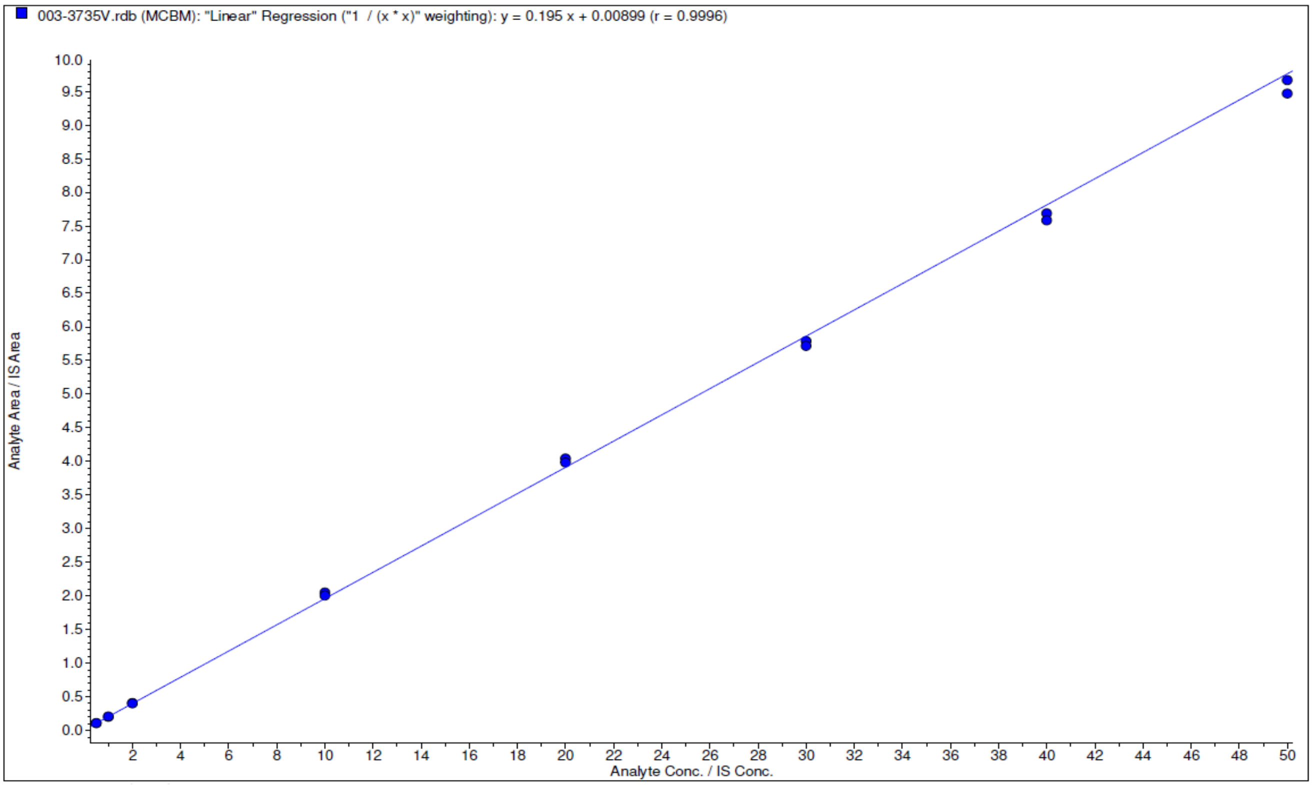

A summary of the analytical method is shown below:

Analyte (Abbreviation)	Methocarbamol (MCBM)
Internal Standard (Abbreviation)	Methocarbamol-d_5 (MCBM-d_5)
Matrix	Human EDTA K_2 plasma
Sample Volume	50 µL
Sample Pre-treatment	Not required
Extraction Method	Protein precipitation extraction
Analytical Method	LC/MS/MS
Quantitation Method	Peak area ratio
Calibration Regression	Linear
Weighting Factor	$1/X^2$

A summary of the validation results is shown below

Linearity:	$r \geq 0.9995$
Calibration Curve Range:	0.50 - 50.00 µg/mL
Between-Run Accuracy:	% nominal conc.: 92.73% - 99.00%
Between-Run Precision:	CV (%): 1.64% - 2.34%
Within-Run Accuracy:	Assay 1: % nominal conc.: 95.11% - 98.95%
	Assay 2: % nominal conc.: 92.44% - 98.43%
	Assay 3: % nominal conc.: 90.61% - 99.62%
Within-Run Precision:	Assay 1: CV (%): 1.02% - 2.94%
	Assay 2: CV (%): 0.89% - 2.01%
	Assay 3: CV (%): 0.58% - 1.88%
Matrix Selectivity:	No significant interferences observed in any of the different tested matrices for methocarbamol

Matrix Factor: *(in normal, haemolyzed and hyperlipidaemic matrices)*	CV (%): 0.72% and 0.60%
Potentially Interfering Drugs:	No effect on analyte quantitation for all the tested drugs: acetaminophen (paracetamol), caffeine, ibuprofen, nicotine, acetylsalicylic acid, salicylic acid, ethinyl estradiol, drospirenone, levonorgestrel, etonogestrel (3-ketodesogestrel), gestodene, norgestimate, norelgestromin and dienogest
Lower Limit of Quantitation (LLOQ):	0.50 µg/mL (S/N = 204.12)
Dilution Integrity Accuracy:	% nominal conc.: 99.61% and 99.56%
Dilution Integrity Precision:	CV (%): 0.88% and 0.76%
Recovery of Analyte:	94.13%, 92.89% and 94.57%
Recovery of Internal Standard:	101.68%
Autosampler Carryover:	No significant carryover observed
Reinjection Reproducibility Accuracy at Room Temperature:	% nominal conc.: 92.55% - 99.86%
Reinjection Reproducibility Precision at Room Temperature:	CV (%): 0.80% - 1.62%
Sample Collection and Handling Stability at Room Temperature: *(after 60 minutes in human EDTA K_2 whole Blood)*	Did not meet acceptance criteria<br>Stability proved after 60 minutes at 4 °C
Sample Collection and Handling Stability at 4 °C: *(after 60 minutes in human EDTA K_2 whole Blood)*	% change: 6.52% and -1.17%

Post-Preparative Stability at Room Temperature:<br>(*after 168 hours*)	% nominal conc.: 103.83% and 99.96%
Freeze and Thaw Stability at -20 ºC:<br>(*after 4 cycles*)	% nominal conc.: 101.58% and 103.89%
Freeze and Thaw Stability at -80 ºC:<br>(*after 4 cycles*)	% nominal conc.: 107.09% and 102.03%
Short-Term Stability of Analyte in Matrix at Room Temperature:<br>(*after 26 hours*)	% nominal conc.: 102.71% and 105.41%
Short-Term Stability of Analyte in Matrix at 4 ºC:<br>(*after 26 hours*)	% nominal conc.: 106.97% and 102.19%
Short-Term Stability of Analyte in Stock Solution at Room Temperature:<br>(*after 47 hours*)	% change: -2.28%
Short-Term Stability of Analyte in Working Solutions at Room Temperature:<br>(*after 51 hours*)	% change: 5.43%
Short-Term Stability of Internal Standard in Stock Solution at Room Temperature:<br>(*after 47 hours*)	% change: -4.10%
Short-Term Stability of Internal Standard in Secondary Solution at Room Temperature:<br>(*after 47 hours*)	% change: -2.21%
Long-Term Stability of Analyte in Matrix at -20 ºC:<br>(*after 10 days*)	% nominal conc.: 106.72% and 104.90%

Long-Term Stability of Analyte in Matrix at -80 ºC:<br>*(after 10 days)*	% nominal conc.: 106.11% and 104.19%
Long-Term Stability of Analyte in Stock Solution at -20 ºC:<br>*(after 14 days)*	% change: 4.13%
Long-Term Stability of Analyte in Working Solutions at -20 ºC:<br>*(after 13 days)*	% change: -1.40%
Long-Term Stability of Internal Standard in Stock Solution at -20 ºC:<br>*(after 14 days)*	% change: 2.95%
Long-Term Stability of Internal Standard in Secondary Solution at -20 ºC:<br>*(after 14 days)*	% change: -0.01%

Additionally, to the full validation presented above a Partial Validation 1 was also performed:

- To extend long-term stabilities of analyte and internal standard in stock solutions at -20 ºC (349 days)
- To extend long-term stability of analyte in working solutions at -20 ºC (50 days)
- To extend long-term stability of internal standard in secondary solution at -20 ºC (49 days)
- To extend long-term stabilities of analyte in matrix at -20 ºC and -80 ºC (347 days).

Partial Validation 1 summary results are presented below:

Long-Term Stability of Analyte in Matrix at -20 ºC: *(after 347 days)*	% nominal conc.: 109.95 and 108.06%
Long-Term Stability of Analyte in Matrix at -80 ºC: *(after 347 days)*	% nominal conc.: 107.63 and 108.17%
Long-Term Stability of Analyte in Stock Solution at -20 ºC: *(after 349 days)*	% difference: 6.97%
Long-Term Stability of Analyte in Working Solutions at -20 ºC: *(after 50 days)*	% difference: -1.84%
Long-Term Stability of Internal Standard in Stock Solution at -20 ºC: *(after 349 days)*	% difference: 4.49%
Long-Term Stability of Internal Standard in Secondary Solution at -20 ºC: *(after 49 days)*	% difference: 4.12%

This method met the acceptance criteria for all the validation parameters evaluated, demonstrating an acceptable performance and is suitable for the determination of methocarbamol in human EDTA K2 plasma using LC/MS/MS.
